# Matrine@chitosan-D-proline nanocapsules as antifouling agents with antibacterial properties and biofilm dispersibility in the marine environment

**DOI:** 10.3389/fmicb.2022.950039

**Published:** 2022-07-22

**Authors:** Xiangping Hao, Weilu Yan, Jingzhi Yang, Yun Bai, Hongchang Qian, Yuntian Lou, Pengfei Ju, Dawei Zhang

**Affiliations:** ^1^National Materials Corrosion and Protection Data Center, Institute for Advanced Materials and Technology, University of Science and Technology Beijing, Beijing, China; ^2^Belt and Road Initiative (BRI) Southeast Asia Network for Corrosion and Protection, Ministry of Education (MOE), Shunde Graduate School of University of Science and Technology Beijing, Foshan, China; ^3^Shanghai Aerospace Equipment Manufacturer, Shanghai, China; ^4^Beijing Advanced Innovationation Center for Materials Genome Engineering, University of Science and Technology Beijing, Beijing, China

**Keywords:** antibacterial, biofilm dispersal, pH-responsive, matrine, D-proline, antifouling coatings, marine environment

## Abstract

Antifoulants are the most vital substances in antifouling coatings to prevent marine organisms from colonizing the undersea substrate surfaces. In addition to antibacterial performance, inhibition of biofilm formation is an important criterion for antifouling coatings. In this study, we synthesized pH-responsive matrine@chitosan-D-proline (Mat@CS-Pro) nanocapsules of about 280 nm with antibacterial properties and biofilm dispersibility. The prepared Mat@CS-Pro nanocapsules exhibited high-level antibacterial properties, reaching about 93, 88, and 96% for *E. coli, S. aureus*, and *P. aeruginosa*, respectively. Such nanocapsules can cause irreversible damage to bacteria and cause them to lose their intact cell structures. Moreover, Mat@CS-Pro nanocapsules also possessed outstanding dispersal biofilm performances, in which the biofilm thickness of *E. coli, S. aureus*, and *P. aeruginosa* was decreased by 33, 74, and 42%, respectively, after 3 days of incubation. Besides, the Mat@CS-Pro nanocapsules had remarkable pH-responsive properties. As the environmental pH became acidic, the nanocapsules swelled to about 475 nm and the released concentration could reach 28.5 ppm after immersion for 10 h but maintained a low releasing rate in pH 8 conditions.

## Introduction

Marine biofouling is a worldwide issue exerting a significant impact on our daily lives, especially the economic losses caused by shipping and the safety risks of engineering equipment. At present, the most traditional and prevalent approach is to apply antifouling paint. Antifoulants are active ingredients in antifouling coatings; they have toxicity and bactericidal activity and can be released from coatings to prevent marine organisms from colonizing the surface of submarine substrates. After tributyltin was forbidden by the International Maritime Organization, copper-based and zinc-based inorganic antibacterial agents dominated the marketing (Alzieu, 2000; Amara et al., 2018). Moreover, as most natural organic antifoulants have eco-friendly features, they have been developed rapidly in recent years, such as capsaicin and camptothecin (Hao et al., 2020, 2022a). In designing antifoulants, researchers have focused on how to kill the fouling organisms and inhibit the growth and reproduction of organisms near the surfaces of the submarine substrate. However, dispersing mature biofilm and inhibiting biofilm formation on substrate surfaces are also vital problems to be considered, as biofilms frequently serve as breeding grounds for other fouling creatures and accelerate fouling processes (Lejars et al., 2012; Qian et al., 2019; Hao et al., 2022b).

D-amino acids as enantiomers of L-amino acids are generated by racemase (Yoshimura and Esak, 2003; Pollegioni et al., 2020). Due to their degradability and non-toxicity, they are widely used in pharmaceutical synthesis, analysis of enzyme structure and functions, and bactericide applications (Asano and Lübbehüsen, 2000; Hao et al., 2017; Kano et al., 2019; Huang et al., 2020; Williams et al., 2021). Notably, as a new type of biofilm dispersant, D-amino acids have received significant research interest in the past decade. Kolodkin-Gal et al. (2010) discovered that before biofilm disassembly, *Bacillus subtilis* produced a factor that prevented biofilm formation and could break down existing biofilms. The factor consisted of D-leucine, D-methionine, D-tyrosine, and D-tryptophan. As a potential class of biofilm dispersal materials, D-amino acid not only disperses mature biofilms but also prevents the formation of biofilms of bacteria such as *Staphylococcus aureus* (*S. aureus*) and *Pseudomonas aeruginosa* (*P. aeruginosa*). For example, Chang et al. (2022) revealed that the prepared inhalation powders synthesized by ciprofloxacin and D-amino acids (such as D-methionine, D-tryptophan, and D-phenylalanine) in a mass ratio of 7:3 can facilitate *P. aeruginosa* biofilm removal. Warraich et al. (2020) proposed that the D-aspartic acid and D-glutamic acid can improve the solubility of the ciprofloxacin and enhance the antibacterial effects and biofilm dispersibility of *S. aureus*. The results showed that the synergistic effect of the D-amino acid and ciprofloxacin can achieve 97% of inhibition effect and 98% of dispersal biofilm ability. They can not only disperse established biofilms but also inhibit the formation of new biofilms.

To date, more than 10 types of D-amino acids, including D-glutamic acid, D-aspartic acid, D-leucine, D-methionine, D-tyrosine, D-tryptophan, D-tryptophan, D-phenylalanine, D-proline, and D-cysteine, have been demonstrated to possess biofilm dispersion effects (Ramón-Peréz et al., 2014; Huang et al., 2020; Warraich et al., 2020; Hao et al., 2021a; Chang et al., 2022). As a typical D-amino acid, D-proline also exhibited biofilm dispersibility. Ramón-Peréz et al. (2014) demonstrated that when D-proline was mixed with D-methionine and D-phenylalanine, the biofilm formed by *Staphylococcus epidermidis* was disrupted, thereby reducing the risk of infection of contact lenses. Hochbaum et al. (2011) evaluated the effects of L-proline and D-proline on the structural features of *Escherichia coli* (*E. coli*) K-12 cells under high salinity conditions. The results showed that the D-proline had almost no effect as a compatible solute and even inhibited glucose uptake of *S. aureus* K-12 under the conditions of 0.5 M NaCl, which means the D-proline could trigger the dysfunction of bacterial cells and affect bacterial reproduction. Hence, it could be a feasible idea to introduce D-proline in the preparation of antifoulants to confer their desperate biofilm abilities.

At present, researchers have developed pH-responsive, enzyme-responsive, and temperature-responsive materials to decrease the releasing rate of the antibacterial agents to prolong the serving life (Zhang et al., 2020; Aytac et al., 2021; Hao et al., 2021b; Huang et al., 2022). The pH-responsive technology has been a popular approach to consider when synthesizing antifoulants because the ambient pH of the environment near the surface of underwater substrates changes after bacterial proliferation. Chitosan (CS) as an inexpensive, non-toxic material can be used to encapsulate antibacterial drugs and prepare pH-responsive antibacterial nanocapsules. The pH-controlled releasing behaviors of the nanocapsules were achieved *via* the protonation and deprotonation under acid and alkaline conditions of the amino group of CS, respectively (Wang et al., 2018; Hao et al., 2020; Hamedi et al., 2022). The oil solubility drugs such as capsaicin, ibuprofen, allicin, and leaf-adhesive avermectin have been encapsulated in CS and exhibited pH-controlled releasing behaviors (Hao et al., 2019, 2022c; Chen et al., 2021; Lei et al., 2021). Matrine is widely used in traditional herbal medicine and has recently been approved for the treatment of various anti-cancer, viral and parasitic, neuropathy, and applied agriculture fields (Li et al., 2019, 2020, 2021). Moreover, it also possesses outstanding antimicrobial effects (Jia et al., 2019; Sun et al., 2019; Zhou et al., 2020). For example, Zhou et al. (2020) demonstrated that the incorporated matrine improved the antibacterial activity of konjac glucomannan/fish gelatin hydrogel, and the results showed that the inhibitory zones for *E. coli* and *S. aureus* were about 12.0 and 11.5 mm when the matrine concentration was 40 mg/ml. Hence, matrine may be used as an environmentally friendly candidate to replace biocides in the preparation of antifoulants.

Herein, a multifunctional pH-responsive antifoulant was prepared by combining the biofilm dispersibility of D-proline with the antibacterial effect of matrine. We prepared matrine@chitosan (Mat@CS) nanocapsules by microemulsion methods initially, followed by loading the D-proline on the surfaces of nanocapsules to obtain matrine@chitosan-D-proline (Mat@CS-Pro) multifunctional nanocapsules. The morphology and composition of the nanocapsules were evaluated by transmission electron microscopy (TEM) and Fourier transform infrared spectroscopy (FTIR). The antibacterial properties of the nanocapsules were detected by the colony counting method, and the morphology of bacteria after incubation with and without Mat@CS-Pro nanocapsules was observed by scanning electron microscopy (SEM). The biofilm dispersibility of the Mat@CS-Pro nanocapsules was verified by SEM and the Live/dead® BacLight TM Bacterial Viability Kit. Dynamic light scattering (DLS), UV-Visible absorption spectrum (UV-Vis), and TEM were utilized to analyze the change in diameter of nanocapsules, releasing behaviors of matrine and D-proline at different pH solutions. The pH-responsive antibacterial properties of the Mat@CS-Pro nanocapsules were evaluated by optical density (O.D.) and the plate colony method.

## Experimental section

### Materials

CS with a low viscosity <200 mPa·S, matrine with a purity of 98%, and D-proline with a purity of 99% were purchased from Aladdin Chemistry Co. Ltd. (China). Lecithin with a purity >98% was provided by Shanghai Macklin Biochemical Co. Ltd (China). The 2216E medium powders were purchased from Qingdao Hope Bio-Technology Corporation. Different pH phosphate-buffered saline (PBS) solutions were stored after sterilization. Live/dead® BacLight TM Bacterial Viability Kit (L13152) was obtained from Thermo Fisher Scientific (America). The *E. coli* (ATCC 8739), *S. aureus* (ATCC 6538), and *P. aeruginosa* (MCCC 1A00099) were purchased from the Institute of Microbiology, Chinese Academy of Sciences, Beijing, China.

### Preparation of Mat@CS nanocapsules

Mat@CS nanocapsules were prepared by the microemulsion method (Hao et al., 2020). An amount of 32 mg matrine was completely dissolved in 400 μl of lecithin-contained (12 mg) ethyl alcohol solution. An amount of 10 mg CS was fully dispersed in 20 ml of acetic acid aqueous solution (1 wt. %). These two solutions were mixed together and stirred at 200 rpm for 2 h at room temperature. The free matrine was eliminated by dialysis and evaluated by UV-Vis, and the prepared products were obtained after vacuum freeze-drying.

### Preparation of Mat@CS-Pro nanocapsules

An amount of 40 mg D-proline was dissolved in a 20 ml aqueous solution, followed by adding 20 mg Mat@CS into the solutions and stirring at 200 rpm for 48 h in an ice bath. The free D-proline was eliminated by dialysis in an aqueous solution. The Mat@CS-Pro nanocapsules were collected after vacuum freezing at −50°C for 36 h.

### Antibacterial properties of Mat@CS-Pro nanocapsules

The colony counting method was utilized to analyze the antibacterial properties of the Mat@CS-Pro nanocapsules. *E. coli* and *S. aureus* were selected as representatives of gram-negative and gram-positive bacterial strains, and *P. aeruginosa* was selected as a representative of marine bacteria. The initial concentration of each bacterium was about ~10^8^ CFU/ml. A 1 ml suspension of *E. coli* or *S. aureus* was injected into 50 ml of Luria-Bertani broth (LB) and shaken for 18 h at 37°C. A 1 ml suspension of *P. aeruginosa* was transformed into 50 ml of 2216E medium and inoculated for 18 h at 30°C with shaking. Later, 200 μl of cultivated bacterial suspensions were transformed into 8 ml of LB or 2216E medium containing 2 mg/ml of different antibacterial agents (i.e., CS, matrine, D-proline, Mat@CS nanocapsules, and Mat@CS-Pro nanocapsules). After incubation for another 18 h at 37 or 30°C, each 20 μl bacterial suspension diluted with physiological saline was spread on a solid medium plate, incubating overnight at the corresponding temperature conditions. The results of the bacteriostasis were calculated by the following equation (Chang et al., 2022):


(1) $$\begin{array}{r} B_{R}=\frac{AB}{A}\times 100\% \end{array}$$

where A is the count of the bacterial colonies of the control group, and B is the count of the bacterial colonies of the treatment groups.

### Biofilm dispersibility of Mat@CS-Pro nanocapsules

The 316L stainless steel coupons (1 cm × 1 cm) were used for the biofilm dispersibility of the prepared Mat@CS-Pro nanocapsules. The specimens were abraded to 1,200 grit with silicon carbide paper, followed by cleaning with acetone and ethanol in an ultrasonic bath and dried with N_2_. Ultraviolet light was used to sterilize the metal coupons. Each 1 ml of *E. coli* or *S. aureus* suspension was injected into 50 ml of LB shaking at 120 rpm for 18 h at 37°C. A volume of 1 ml of *P. aeruginosa* suspension was transformed into 50 ml of 2216E medium and inoculated at 30°C for 18 h. Each coupon was taken into a 24-well plate, followed by injecting a 4 ml of LB medium (contained 2 mg/ml Mat@CS or Mat@CS-Pro nanocapsules) and 100 μl of the bacterial suspension. After stationary cultivation for 3 days at 37°C or 30°C, the samples were taken out and washed with deionized water to remove the free-floating bacteria on the metal surfaces. The bacteria were fixed with 2.5% (v/v) glutaraldehyde phosphate buffer solution (PBS, pH 7.4) for 2 h. Subsequently, each coupon was washed three times with PBS and three times with deionized water and dehydrated with gradient ethanol of 30, 50, 70, 90, and 100% (v/v) for 15 min. After sputtering specimens with gold, the bacterial films on metal surfaces were investigated by SEM.

Furthermore, the biofilm dispersibility of the Mat@CS-Pro nanocapsules was analyzed using a Live/dead® BacLight TM Bacterial Viability Kit. Each coupon was immersed in different bacterial suspensions containing nanocapsules and incubated for 3 days. Afterward, each sample was washed with deionized water to remove the free-floating bacteria on the metal surfaces and put into a clean 24-well plate, dyed for 20 min in a dark place.

### pH-responsive properties of Mat@CS-Pro nanocapsules

The Mat@CS-Pro nanocapsule suspension (2 mg/ml) was placed in a dialysis bag, immersed in pH 5, 6, 7, and 8 PBS solutions, respectively, for 12 h. The size of the Mat@CS-Pro nanocapsules was detected by DLS and TEM after treatment in different pH PBS solutions. The dialysis solution was measured using UV-Vis, and the peak at about 220 nm represented matrine (Peng et al., 2014).

### pH-responsive antibacterial properties of Mat@CS-Pro nanocapsules

The colony plate method was utilized to evaluate the pH-responsive antibacterial properties of Mat@CS-Pro nanocapsules. Notably, 2 mg/ml of such nanocapsules mediums were prepared, consisting of 4 ml of different pH PBS solutions (pH 5, 6, 7, and 8) and 4 ml LB medium (or 2216E). Each 200 μl bacterial suspension was inoculated into an 8 ml culture medium and incubated for 4 h at either 37 or 30°C. A 20 μl diluted bacterial suspension was spread on the top of the solid medium and cultivated at the corresponding temperature for 18 h. After being cultivated for 4 h, the O.D. value of each bacterial suspension was detected using UV-Vis at 600 nm as a reference.

### Characterization

The morphology of the Mat@CS and Mat@CS-Pro nanocapsules was demonstrated by TEM (FEI Tecnai F20, America). The FTIR VERTEX70 was produced by Burker (Germany). The size and zeta potential of the Mat@CS-Pro nanocapsules were detected using Zetasizer Nano ZS ZEN3600 (Malvern Instrument, Britain). The morphology of the bacteria was observed using SEM (TESCAN MIRA, Czech Republic). The UV-Vis was used by UH5300 (Hitachi, Japan). The inverted phase-contrast microscope (IPCM, Lacia, TCS SP8, Germany) was applied to determine the fluorescent assay.

## Results and discussion

### Characterization of the prepared Mat@CS-Pro nanocapsules

**Figure 1:**
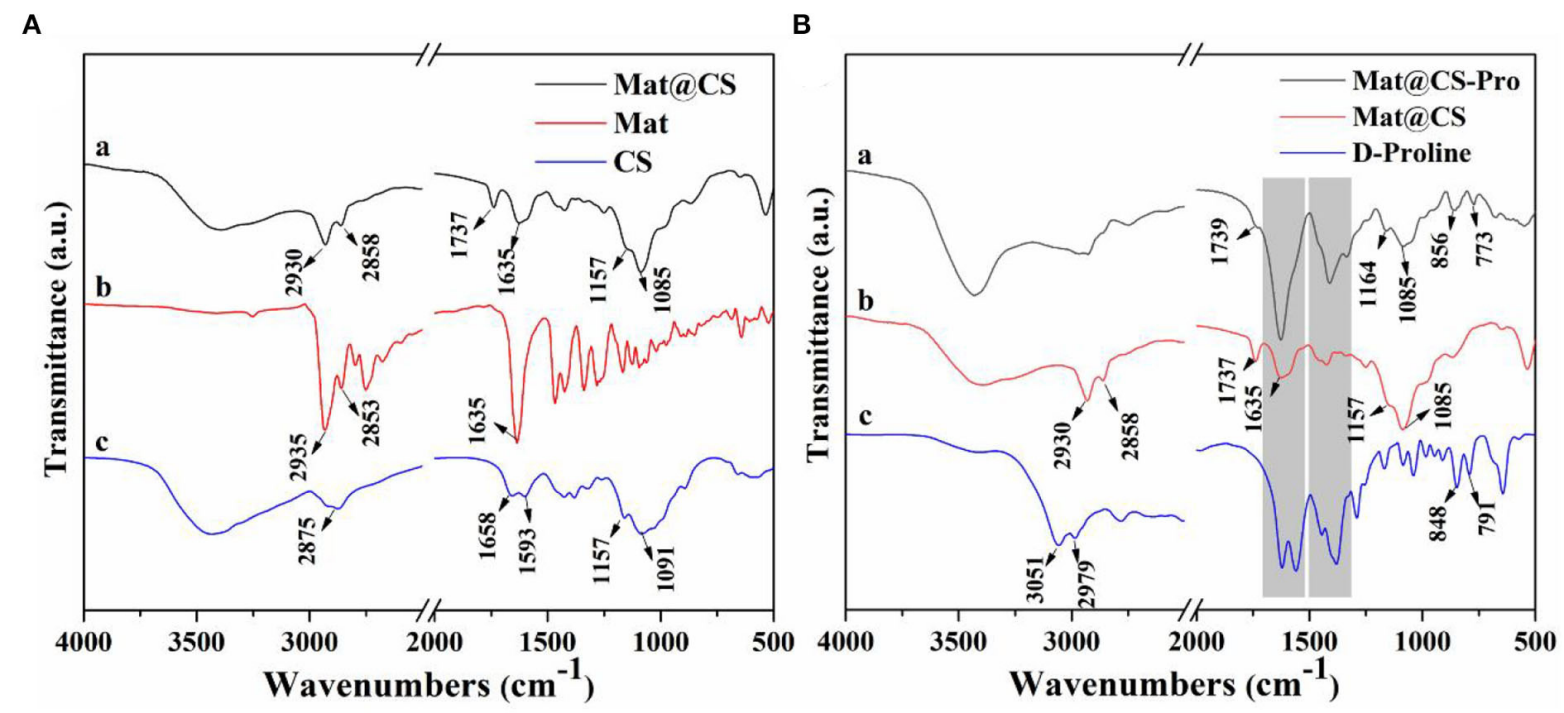
FTIR spectra of the CS, matrine, and Mat@CS nanocapsuels **(A)**; FTIR spectra of D-proline, Mat@CS nanocapsules, and Mat@CS-Pro nanocapsuels **(B)**.

**Figure 2:**
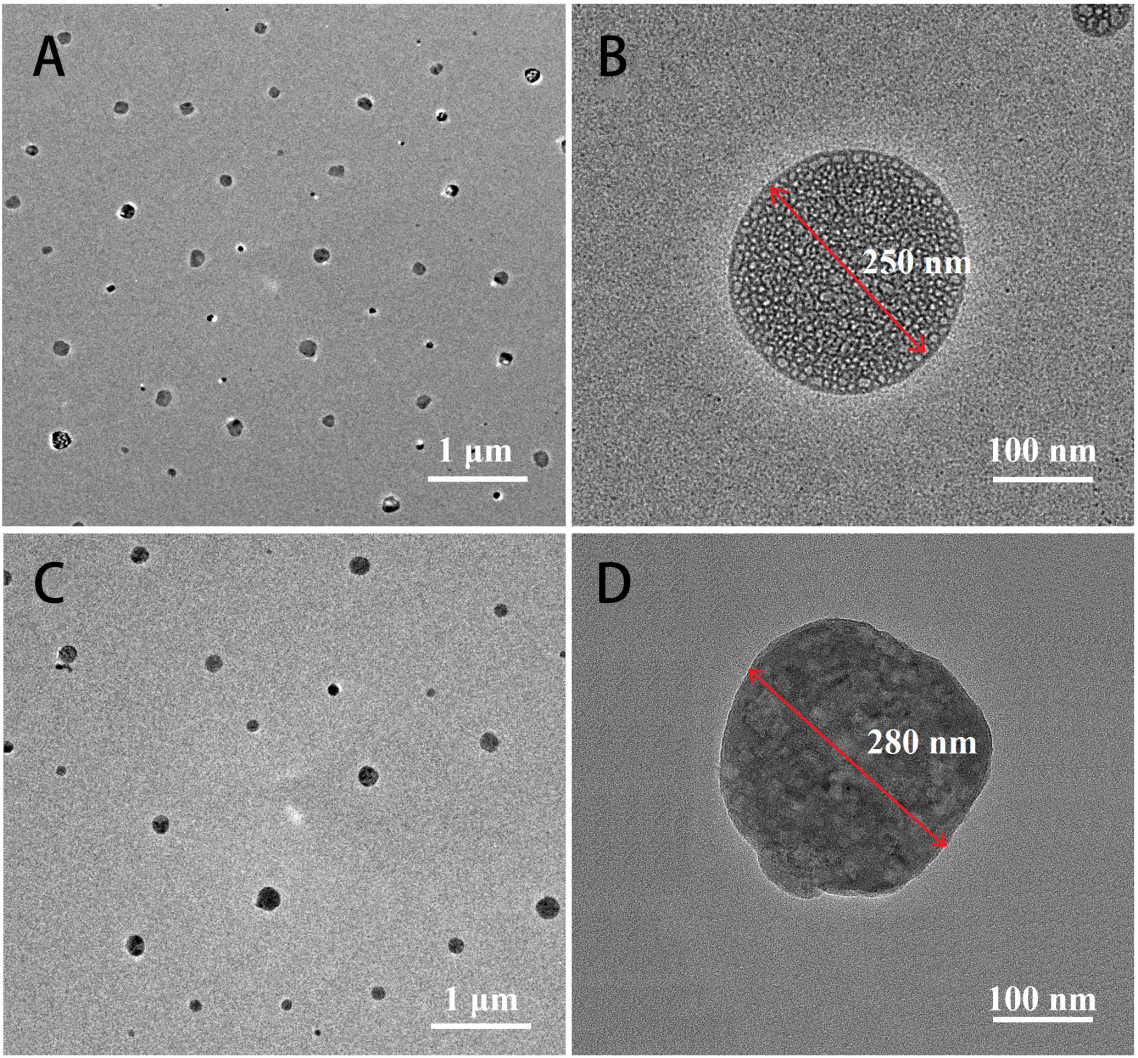
TEM images of Mat@CS **(A,B)** and Mat@CS-Pro nanocapsules **(C,D)**.

The FTIR of the CS, matrine, and microemulsion products is shown in Figure 1A. For the CS spectrum, the broad peak located at 3,500–3,200 cm^−1^ belongs to the N-H and O-H stretching vibration (Hao et al., 2021b). The 1,157 and 1,091 cm^−1^ peaks are attributed to C-O-C stretching vibrations (Ganji and Abdekhodaie, 2008). The 1,658 and 1,593 cm^−1^ peaks are attributed to amino groups of CS. On the matrine spectrum, the 2,935 and 2,853 cm^−1^ peaks belong to the C-H stretching vibration of CH_2_, and the 1,635 cm^−1^ peak is attributed to the C=O stretching vibration (Wu and Yin, 2022). The characteristics of the product spectrum were like the CS spectrum. Specifically, the broad peak around 3,500–3,200 cm^−1^ belongs to N-H and O-H stretching vibrations appears in the spectrum a. Besides, the peak at 1,157 cm^−1^ on the spectrum of emulsion products is associated with C-O-C stretching vibration, and the 1,085 cm^−1^ peak (C-O-C stretching vibration) in the product spectrum corresponds to the 1,091 cm^−1^ peak on the CS spectrum, indicating the CS exists in this product. Meanwhile, correlative characteristic peaks of the matrine can also be found in this spectrum a. The strength of the 1,635 cm^−1^ peak (C=O stretching vibration) in the spectrum a is weaker than that in the matrine spectrum. The peaks belonging to C-H stretching vibration also appear at 2,930 and 2,858 cm^−1^, and the 2,875 cm^−1^ peak on the CS spectrum (C-H stretching vibration) is covered by these two peaks. It means that the matrine also exists in these nanocapsules. Moreover, the new peak located at 1,737 cm^−1^ indicates that the H-bond is existing between CS and matrine (Wang et al., 2018). Combined with the TEM results shown in Figures 2A,B, the Mat@CS nanocapsules were synthesized successfully.

After D-proline treatment, the product component was evaluated using FTIR, and the results are shown in Figure 1B. For the spectrum of D-proline, the 3,051 and 2,979 cm^−1^ peaks belong to the asymmetric stretching and symmetric stretching vibrations of the C-H bond (Mary et al., 2009). The peaks located at 1,600~1,450 cm^−1^ (on the D-proline spectrum) are related to the skeleton ring vibration, and the strong peaks also appear at similar positions on the spectrum of prepared products. Meanwhile, the peaks at 848 and 791 cm^−1^ on spectrum c are contributed by the rocking vibration of CH_2_, and these peaks (856 and 773 cm^−1^) also appear on the spectrum a (Walton et al., 1970; Mary et al., 2009). The peaks at 1,164 and 1,085 cm^−1^ that are attributed to C-O-C of Mat@CS nanocapsules are also present in the spectrum a. The above results indicate that the D-proline was immobilized on the Mat@CS successfully.

The structure and morphology of the surface of the preprepared nanocapsules before and after D-proline immobilization were detected by TEM. Figures 2A,B demonstrated that the Mat@CS nanocapsules prepared *via* oil/water (O/W) microemulsion had uniform sizes with an average diameter of ~250 nm. After immobilization of D-proline, the nanocapsules did not exhibit the aggregation phenomenon, and their diameter was slightly increased as shown in Figure 2C. However, the morphology of the Mat@CS-Pro was different from the Mat@CS nanocapsules, which showed lots of droplets inside the nanocapsules. According to the zeta potential of CS (37.8 ± 1.1 mV), matrine (−16.9 ± 2.4 mV), and Mat@CS nanocapsules (31.9 mV), the droplets in the nanocapsules should be matrine because the negative surface charge of free matrine droplets is completely covered by cationic CS. Besides, the surface of the Mat@CS nanocapsules was covered with irregularly shaped materials (Figure 2D). Based on the −7.7 ± 1.1 and −5.3 ± 0.3 mV zeta potential profiles of D-proline and Mat@CS-Pro nanocapsules, the negative charged D-proline could be immobilized on the surface of Mat@CS nanocapsules. These results agreed with the FITR result shown in Figure 1. Hence, the D-proline was immobilized on the Mat@CS nanocapsule surface, and the diameter of the Mat@CS-Pro nanocapsules was about 280 nm.

### The antibacterial properties of the Mat@CS-Pro nanocapsules

**Figure 3:**
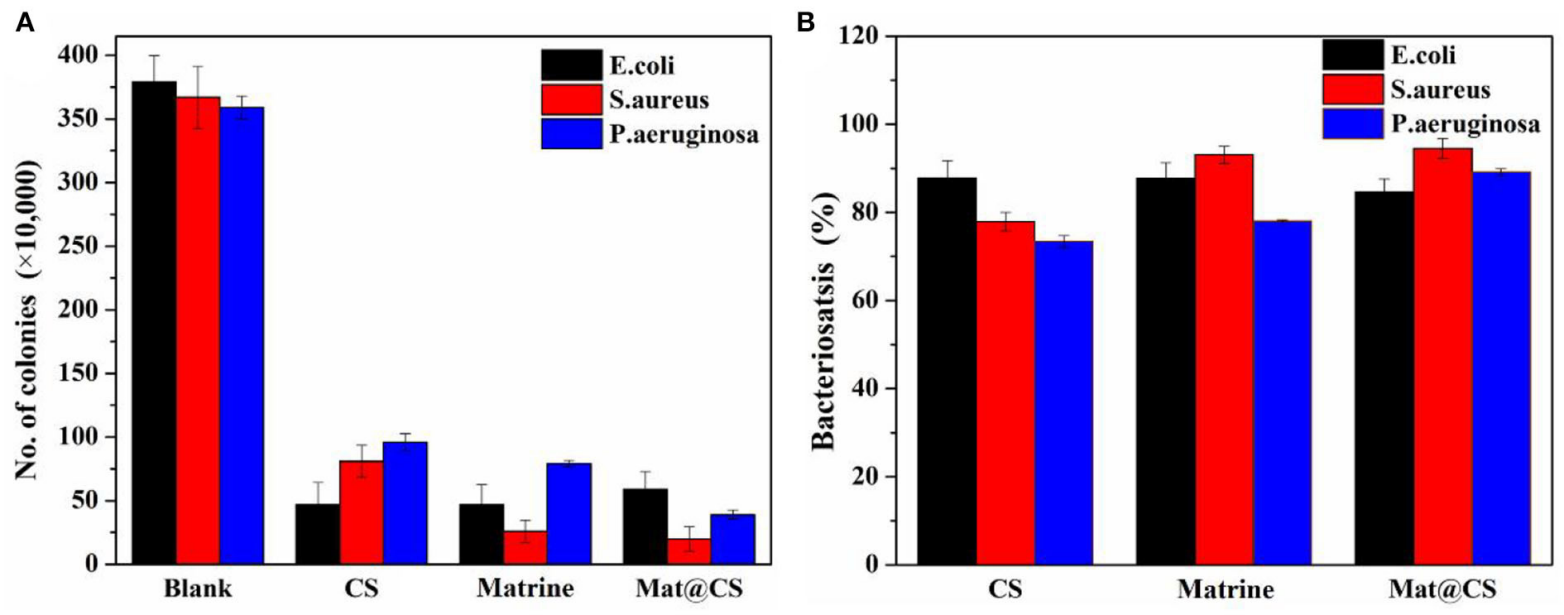
The number of colonies of *E. coli, S. aureus*, and *P. aeruginosa* bacteria after incubation with CS, matrine, and Mat@CS nanocapsules **(A)**; the bacteriostasis of the corresponding counting results **(B)**.

Figure 3 shows the colony counting results and bacteriostasis of the CS, matrine, and Mat@CS nanocapsules against *E. coli, S. aureus*, and *P. aeruginosa*. In Figure 3A, the number of bacteria was reduced to varying degrees after treatment with these three materials compared to the control group (379 CFU), indicating CS, matrine, and Mat@CS nanocapsules all have different degrees of antibacterial effects on the three bacterial strains. Generally, in Figure 3B, the bacteriostases for *E. coli, S. aureus*, and *P. aeruginosa* all surpass 88, 78, and 73%, respectively. Noticeably, as for the pure matrine and Mat@CS nanocapsules, the antibacterial effect against gram-positive bacteria is better than the gram-negative counterparts, and the antibacterial performance is the worst against *P. aeruginosa*. In detail, the bacteriostasis of matrine and Mat@CS against *P. aeruginosa* is about 78 and 89%. However, the corresponding data for *S. aureus* is about 93 and 95%, respectively. In contrast with the effect of pure matrine, Mat@CS nanocapsules exhibit better antibacterial performance; the colonies of *E. coli, S. aureus*, and *P. aeruginosa* are reduced by 8 CFU, 6 CFU, and 39 CFU, respectively. It could be because the direct contact between nanocapsules and bacteria has improved. According to the results shown in Figure 3B, the antibacterial properties of Mat@CS nanocapsules are the best, and the bacteriostatic of *E. coli, S. aureus*, and *P. aeruginosa* is increased to 88, 95, and 89%, respectively.

**Figure 4:**
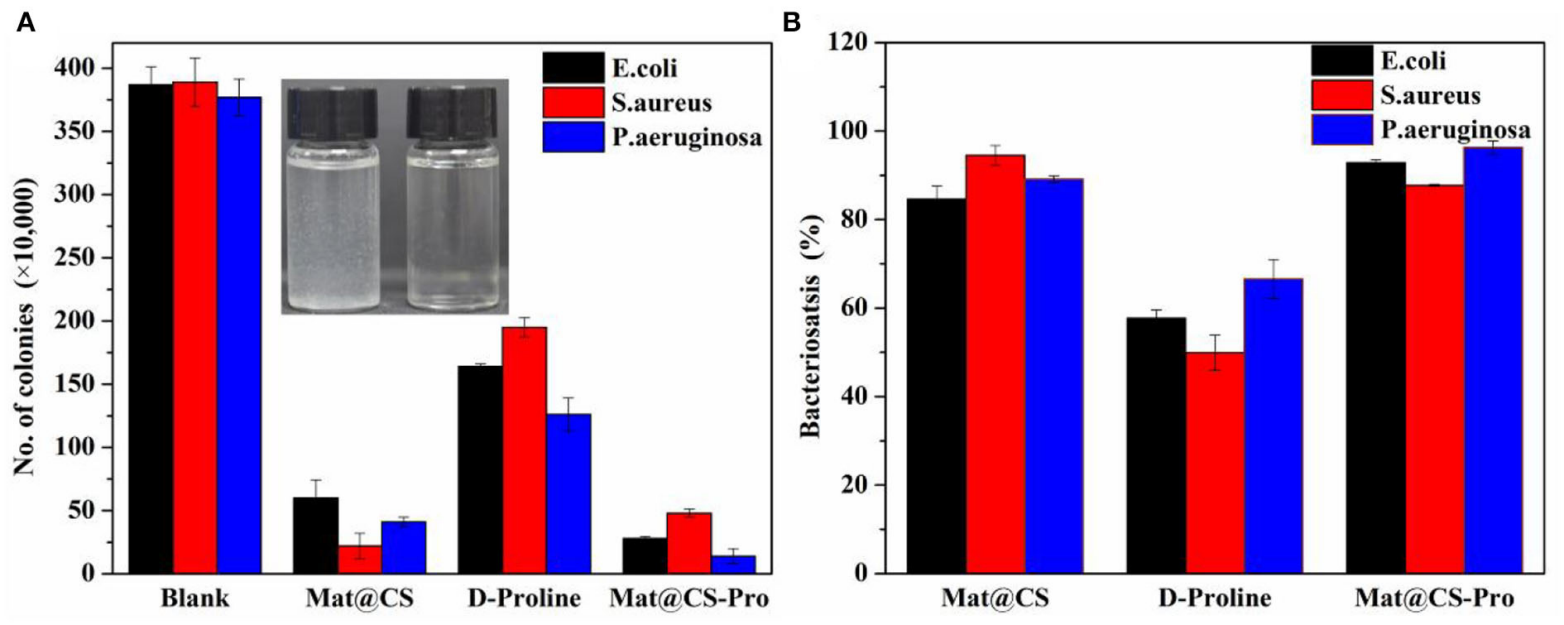
The colony counts of *E. coli, S. aureus*, and *P. aeruginosa* bacteria after treatment with Mat@CS nanocapsules, D-proline, and Mat@CS-Pro nanocapsules **(A)**, and the bacteriostasis of the corresponding colonies counting results **(B)**. The insert image in a is the dispersibility of Mat@CS nanocapsules (left) and Mat@CS-Pro nanocapsules (right) in aqueous solutions.

Furthermore, the antibacterial effects of Mat@CS-Pro nanocapsules were revealed by the colonies counting method. As shown in Figure 4B, the antibacterial performance of the pure D-proline is not satisfactory. The bacteriostasis of D-proline is about 58, 50, and 67% for *E. coli, S. aureus*, and *P. aeruginosa*, respectively. After immobilization of D-proline, the antibacterial performance of Mat@CS nanocapsules regarding *E. coli* and *P. aeruginosa* reaches about 93 and 96%, respectively. However, the antibacterial properties of the nanocapsules against *S. aureus* decreased from 95 to 88% after the introduction of D-proline, which may be due to the poor bacteriostasis of D-proline and the structures of the nanocapsules. After coating D-proline on the Mat@CS surfaces, the releasing behavior of the matrine might be hampered, and the D-proline located on the surfaces could not provide significant bacteriostatic effects, resulting in a slight decrease in the antibacterial properties of Mat@CS-Pro. Besides, after D-proline is immobilized on the Mat@CS nanocapsules, the dispersion is better, which improves the chance of contacting bacterial cells. Based on the positive charge properties of the amino group on CS, the bacterial cell permeability will be changed after contact with CS directly (Kong et al., 2008), especially for Gram-negative strains, which is one of the reasons why the antibacterial performance is improved obversely against such strains after introducing D-proline. Hence, after the introduction of D-proline, the Mat@CS-Pro nanocapsules can almost maintain excellent bacteriostasis for gram-positive bacterial strains and even exert superior performance for gram-negative bacterial strains.

**Figure 5:**
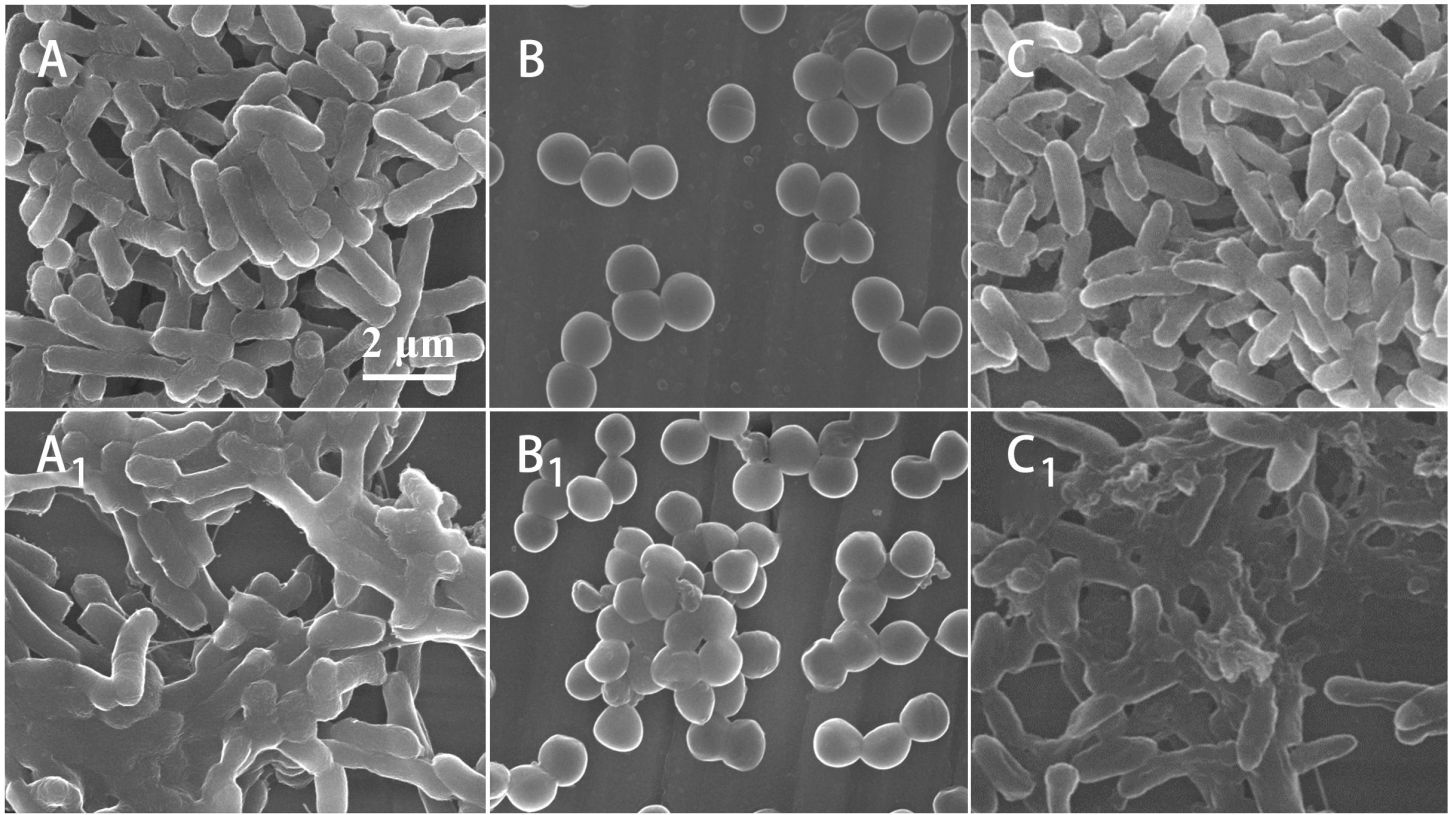
The SEM images of morphologies of *E. coli* **(A)**, *S. aureus* **(B)**, and *P. aeruginosa* **(C)**; morphologies of *E. coli* (A_1_), *S. aureus* (B_1_), and *P. aeruginosa* (C_1_) after treatment by Mat@CS-Pro nanocapsules.

After being treated by Mat@CS-Pro nanocapsules, the morphology of *E. coli, S. aureus*, and *P. aeruginosa* was observed by SEM. In Figure 5, compared with the control group, the bacteria cell membrane almost lost its intact structures, indicating the nanocapsules caused irreversible damage to bacterial cells. Moreover, compared with the morphologies of *S. aureus*, the cell surfaces of *E. coli* and *P. aeruginosa* are coated with lots of organic matter, which proves that the Mat@CS-Pro nanocapsules have a stronger impact on gram-negative bacterial strains than on gram-positive bacterial strains.

### The biofilm dispersibility of the Mat@CS-Pro nanocapsules

**Figure 6:**
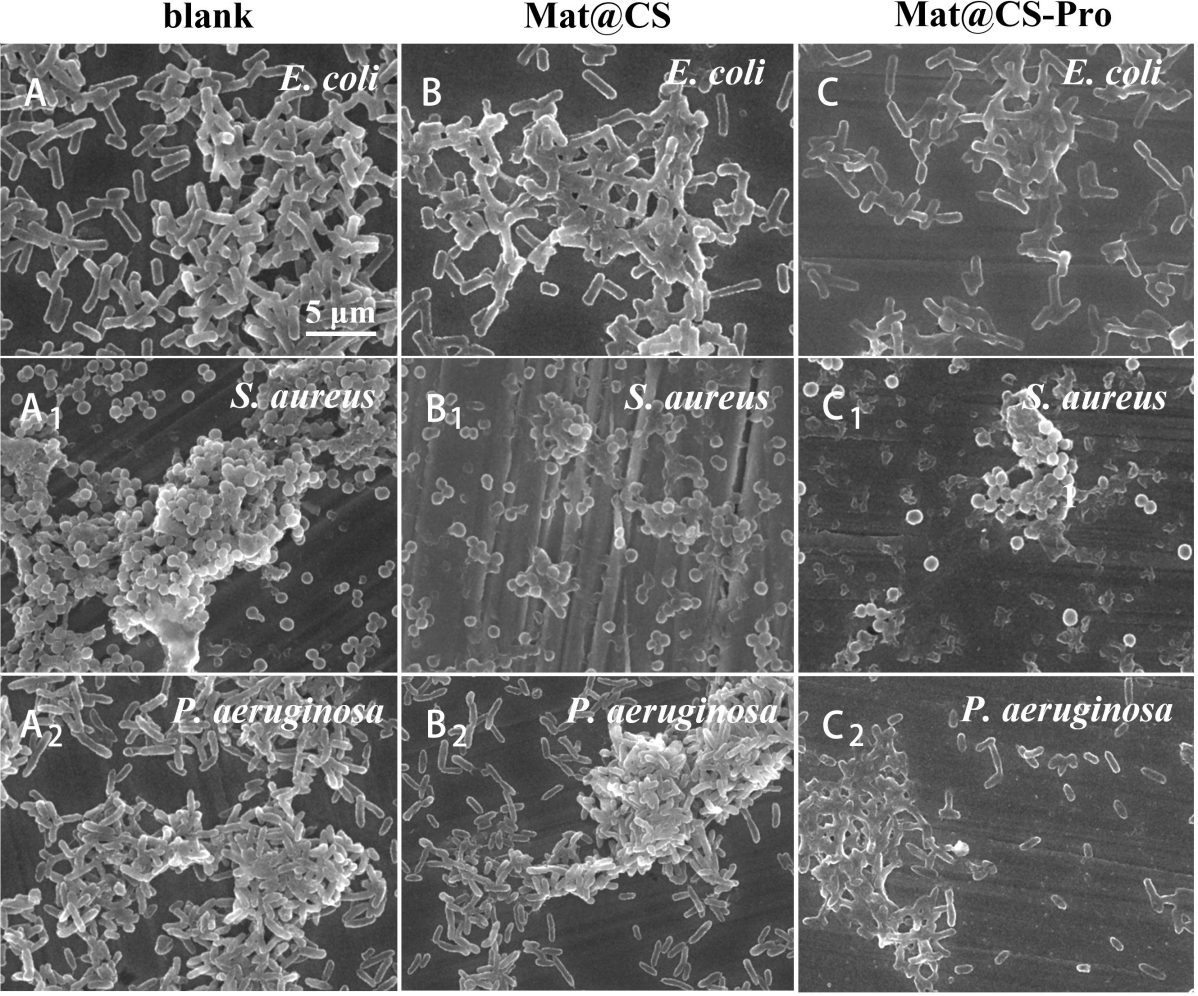
SEM images of *E. coli* **(A–C)**, *S. aureus* (A_1_-C_1_), and *P. aeruginosa* (A_2_-C_2_) biofilms on coupons in cultures with and without Mat@CS and Mat@CS-Pro nanocapsules.

The effects of Mat@CS and Mat@CS-Pro nanocapsules on biofilm formation were evaluated by SEM after incubation for 3 days (Qian et al., 2022). For the control groups, the metal surfaces have the most adherent bacteria and exhibit aggregation (Figures 6A–A_1_). After incubation with Mat@CS nanocapsules, the number of the bacteria cells and the clustering phenomenon are reduced slightly (Figures 6B–B_2_). In Figures 6C–C_2_, after the introduction of D-proline into Mat@CS nanocapsules, adherent bacteria and aggregation effects of bacteria are decreased further, and the adherent bacteria cells in most areas are randomly scattered on the substrate surfaces in the form of single cells. Additionally, the morphologies of bacteria in the aggregative area were significantly different from those in the control groups. When no nanocapsules are present in the medium, the bacteria have intact cell morphology and smooth and clear cell membrane edges. However, when Mat@CS-Pro exists in the system, especially for the aggregative area, the bacteria morphologies are similar to Figures 5A_1_-C_1_, that is, the cell structures tend to fuse together, becoming bacterial clumps (Figures 6C–C_2_).

**Figure 7:**
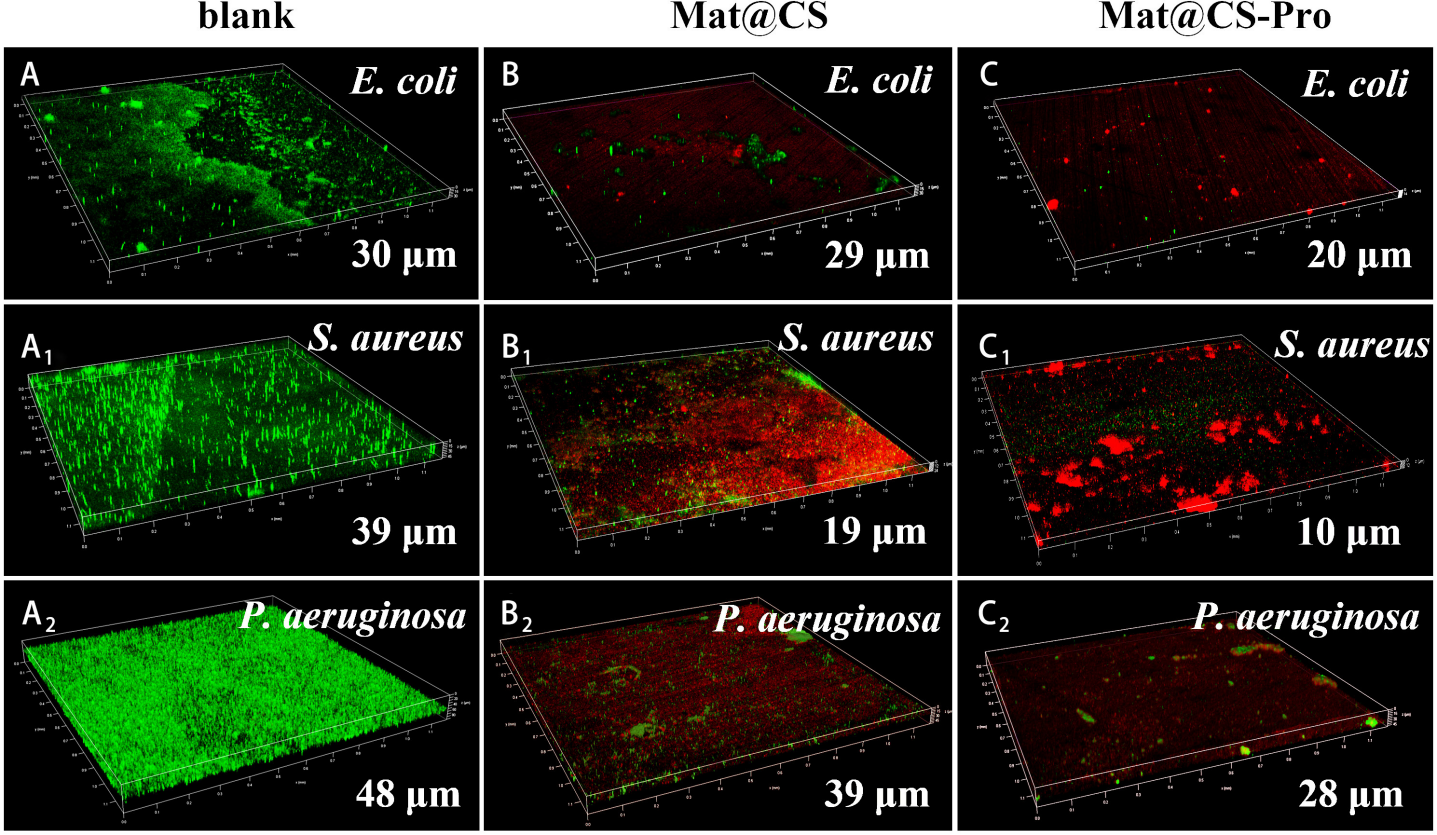
The thickness of biofilms formed by *E. coli* **(A–C)**, *S. aureus* (A_1_-C_1_), and *P. aeruginosa* (A_2_-C_2_) after incubation with Mat@CS and Mat@CS-Pro nanocapsules for 3 days.

The formed biofilms were evaluated by IPCM after 3 days of incubation, and the results are shown in Figure 7. Green and red patterns represented alive and dead bacteria, respectively. For the control group, there are almost all green patterns on the fluorescent images, indicating the 316L stainless steel coupons are not toxic to these three bacteria and the biofilms form well. The thickness of *E. coli, S. aureus*, and *P. aeruginosa* biofilms is 30, 39, and 48 μm, respectively (Figures 7A–A_2_). After incubation with Mat@CS and Mat@CS-Pro nanocapsules, the bacterial activity drops dramatically due to the appearance of many red patterns in the field of vision. In addition, the proportion of green spots is further reduced after the D-proline introduction into the Mat@CS nanocapsules, indicating that the antibacterial effects of Mat@CS-Pro nanocapsules are better than those of the Mat@CS nanocapsules. This agreed with the results in Figure 4. In Figures 7C–C_2_, after incubation with Mat@CS-Pro nanocapsules for 3 days, the thicknesses of formed *E. coli, S. aureus*, and *P. aeruginosa* biofilms are 20, 10, and 28 μm, respectively. The dispersing biofilm ability of the Mat@CS-Pro nanocapsules was significantly improved, and compared with the control group, the thickness of the *E. coli, S. aureus*, and *P. aeruginosa* biofilms decreased by 33, 74, and 42%, respectively. Therefore, based on the existing antibacterial properties, the introduction of D-proline helped to improve the biofilm dispersal performance of the antifouling agents.

### pH-responsive properties of the Mat@CS-Pro Nanocapsules

**Figure 8:**
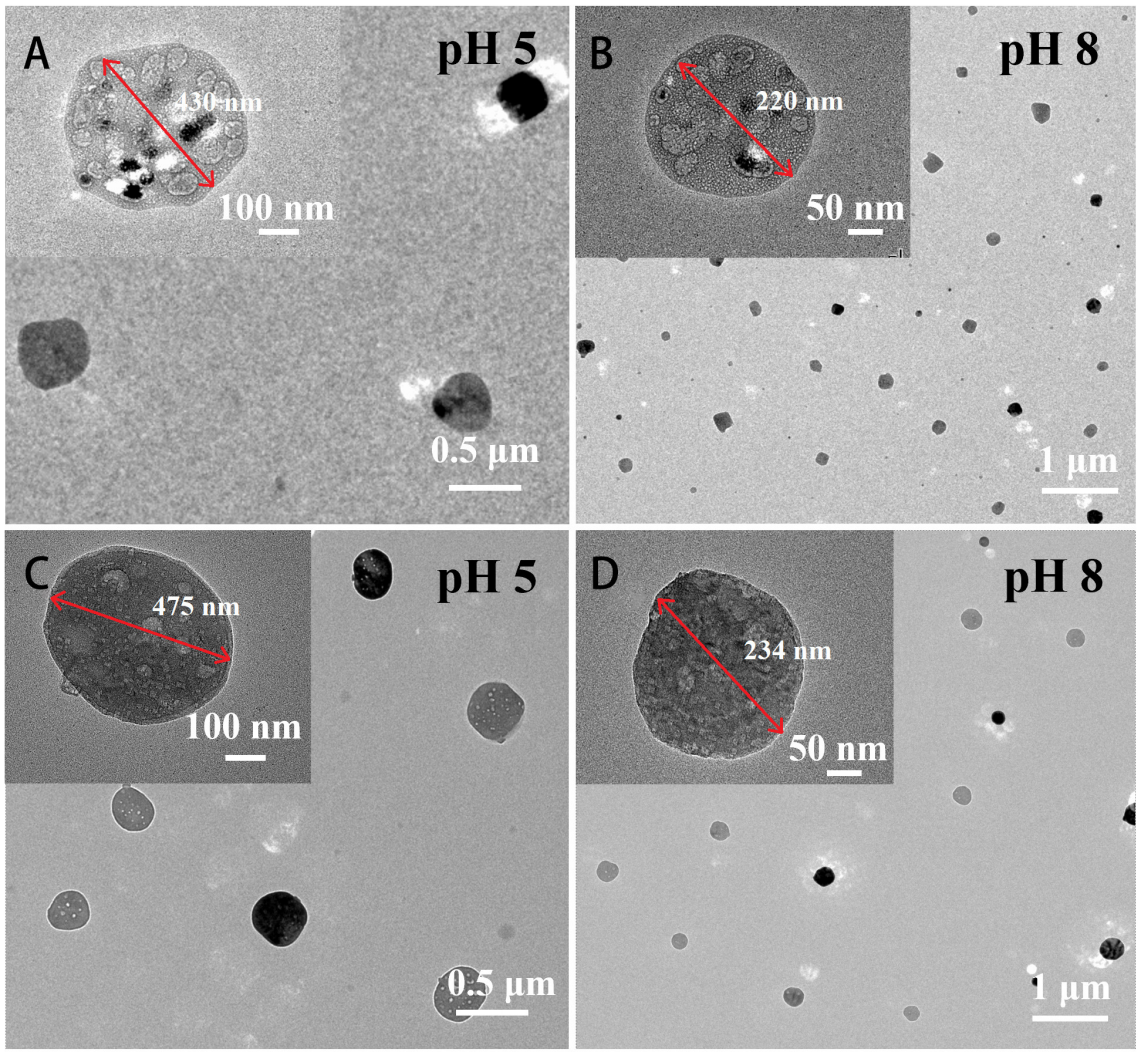
TEM images of Mat@CS nanocapsules after immersion in pH 5 **(A)** and pH 8 **(B)** conditions; Mat@CS-Pro nanocapsules after immersion in pH 5 **(C)** and pH 8 **(D)** conditions.

The structure of the Mat@CS nanocapsule and Mat@CS-Pro nanocapsules after treatment in pH 5 and pH 8 PBS solutions were determined by TEM. Under acid conditions, the –NH_2_ group of CS is converted to ${\text{–NH}}_{3}^{+}$, resulting in positive charges and internal electrostatic repulsion in the system (Hao et al., 2019). Therefore, after immersion in pH 5 conditions, the Mat@CS nanocapsules are swollen from 280 to ~430 nm (Figure 8A). In the marine pH environment (pH 8), the Mat@CS nanocapsules shrink to ~220 nm due to the deprotonation of the amino group of CS as shown in Figure 8B. After immobilization of D-proline, the pH-responsive properties of the nanocapsules are still present. In Figures 8C,D, the diameters of the Mat@CS-Pro nanocapsules are ~475 nm and 234 nm after treatment under alkaline and acid PBS solutions, respectively. The results demonstrated that the Mat@CS-Pro nanocapsules possessed pH-responsive properties, and the diameter of such nanocapsules can change with the ambient pH.

**Figure 9:**
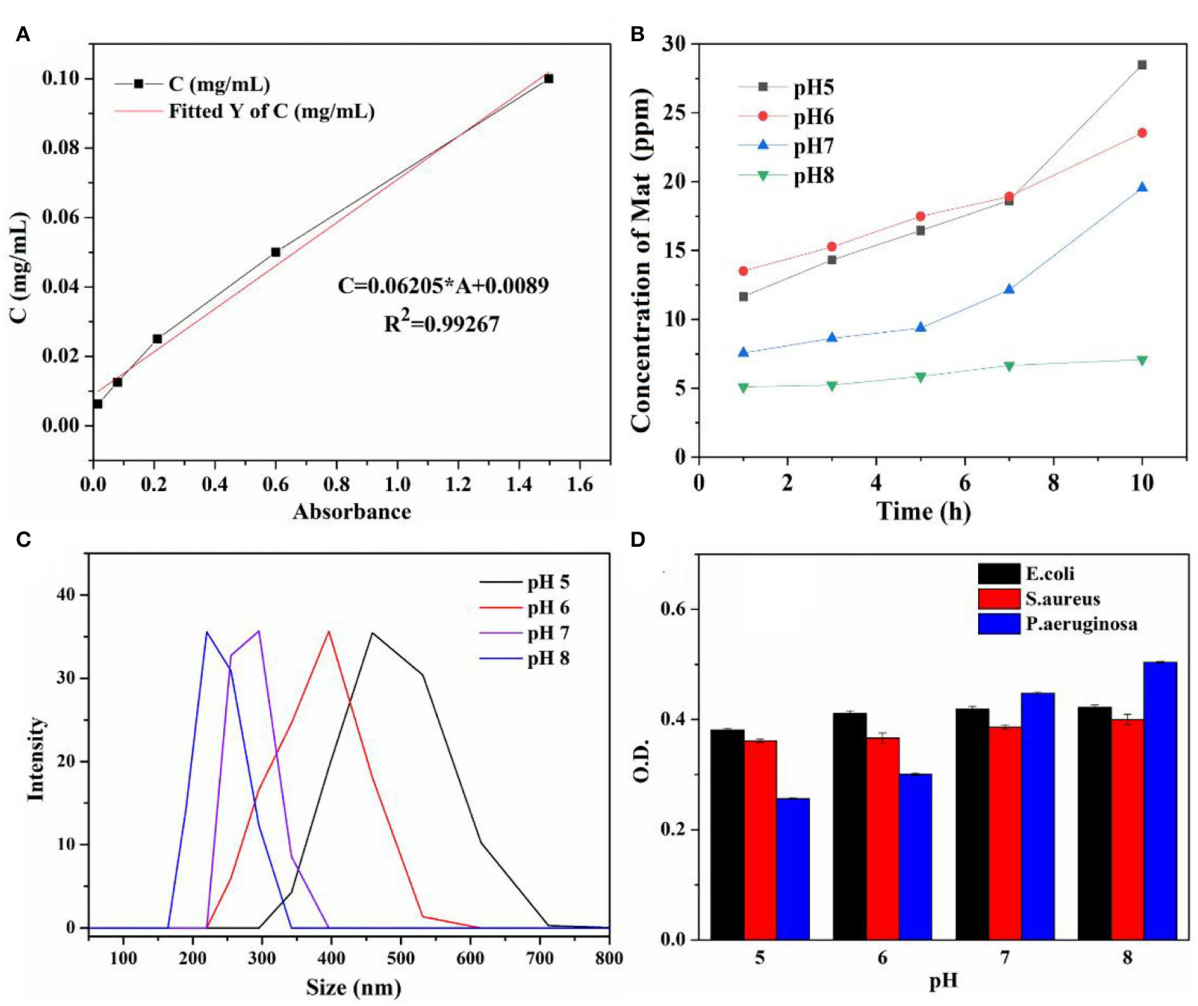
Standard curve of matrine in PBS solutions **(A)**; the concentration of the released matrine from Mat@CS-Pro nanocapsules in different pH PBS solutions for 10 h **(B)**; the diameter of the Mat@CS-Pro nanocapsules after treatment in different pH PBS solutions **(C)**; the O.D. value of three different types of bacterial strains after incubation in different pH LB and PBS solutions (with volume ratio 1:1) containing Mat@CS-Pro nanocapsules for 4 h **(D)**.

The pH-controlled releasing behaviors and mechanisms were determined using UV-Vis and the DLS analysis. The concentrations of the released matrine from Mat@CS-Pro nanocapsules calculated according to the standard curve (Figure 9A) are shown in Figure 9B. The concentration of the released matrine in alkaline conditions is maintained at a low level, from 5.1 ppm at the initial state to 7.1 ppm after 10 h. Meanwhile, the DLS results revealed that the Mat@CS-Pro nanocapsules have the smallest size after immersion in pH 8 PBS solutions, which is ~236 ± 13 nm. In contrast, the released amount of matrine increases continuously in acid solutions, and the concentrations of matrine are ~28.5 and 23.5 ppm after immersion in pH 5 and pH 6 solutions for 10 h, respectively. Compared with the initial state, the released matrine concentrations are increased by 16.8 ppm and 10.3 ppm. In Figure 9C, the diameters of the nanocapsules after treatment in pH 5 and pH 6 solutions are about 478 ± 18 and 396 ± 21 nm, and these results were agreed with Figure 8. Compared with their counterparts in pH 8 conditions, the diameters of the nanocapsules are increased by ~242 and 160 nm, respectively. At pH 7, the concentration of the released matrine shows an upward trend within 10 h. In detail, the concentration of the released matrine slightly increases from 7.6 ppm after 1 h to 9.4 ppm after 5 h and further increases to 10.1 ppm after 10 h. According to the above results, the pH-responsive properties of the Mat@CS-Pro nanocapsules were not limited after the immobilization of D-proline. With the increasing pH of the PBS solution, the releasing behaviors of the matrine and the size of the nanocapsules decrease.

**Figure 10:**
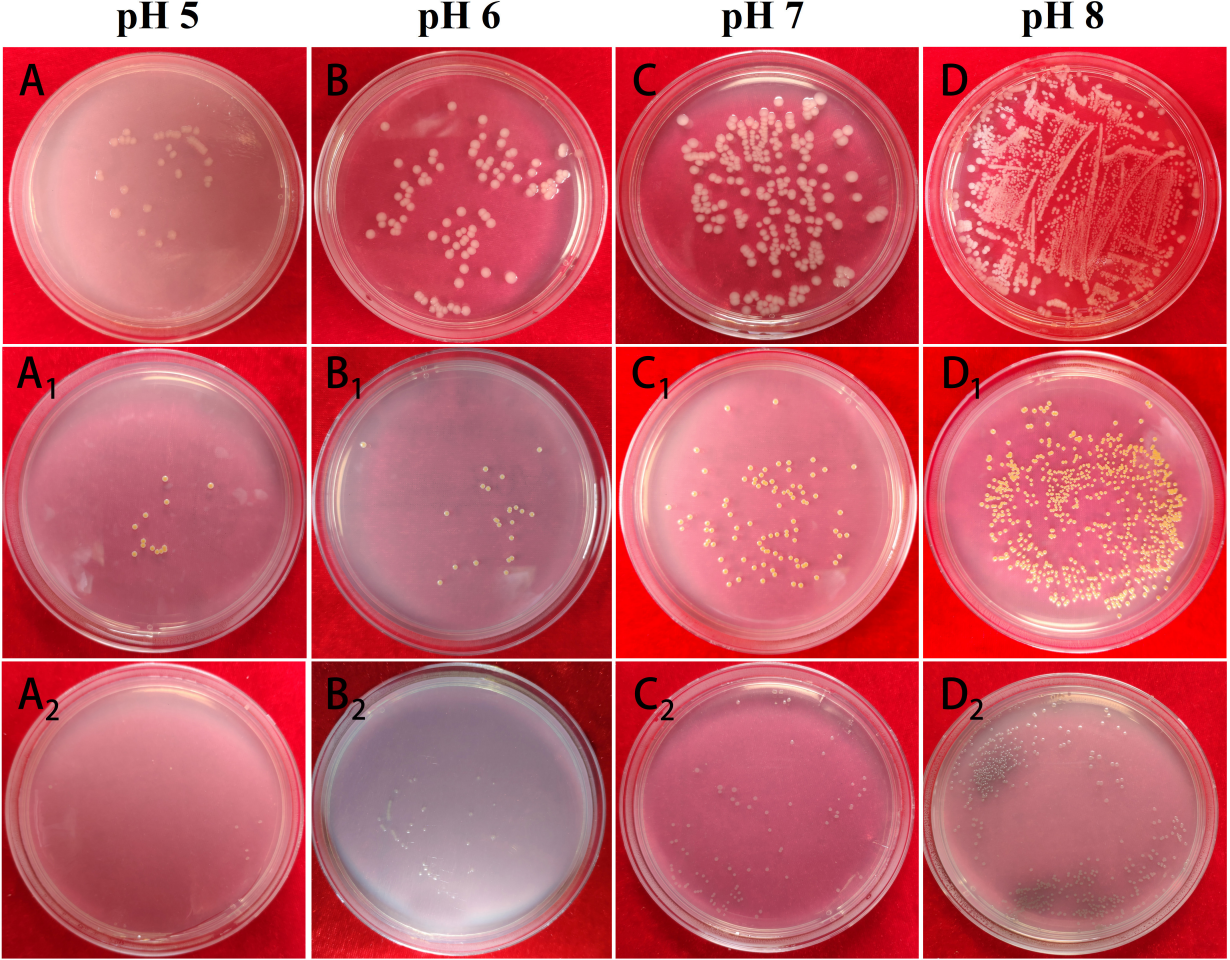
The table colony plate photographs of Mat@CS-Pro against *E. coli* **(A–D)**, *S. aureus* (A_1_-D_1_), and *P. aeruginosa* (A_2_-D_2_) in pH 5, 6, 7, and 8 culture mediums.

The impact of Mat@CS-Pro nanocapsules on the bacterial growth states of *E. coli, S. aureus*, and *P. aeruginosa* in the different pH culture mediums is evaluated by UV-Vis. Bacterial concentration is increased with an increase in O.D. value. In general, Figure 9D illustrates that the O.D. value goes up as the pH of culture mediums increases for these three types of bacterial strains, indicating that as the pH increases, the number of bacteria in the culture medium also increases. Specifically, after incubation at pH 5, 6, 7, and 8 conditions, the O.D. profiles of *E. coli* are ~0.34 ± 0.02, 0.35 ± 0.02, 0.38 ± 0.01, and 0.39 ± 0.02, respectively. In Figures 10A–D, the table colony plate photos results are consistent with the O.D. data, that is, the number of bacterial colonies increases obviously as the environmental pH increases. The O.D. values of *S. aureus* have a similar trend compared with the profiles of *E. coli*, which is about 0.34 ± 0.01, 0.35 ± 0.01, 0.36 ± 0.01, and 0.36 ± 0.01 after incubation at a series of pH culture mediums in order. This result agrees with Figures 10A_1_-D_1_. As for the *P. aeruginosa* group, the O.D. value increased from 0.27 ± 0.01 in the pH 5 condition to 0.45 ± 0.05 in the pH 8 condition. In Figures 10A_2_-D_2_, there are more colonies scattered on the solid mediums after reproduction in pH 7 and 8 conditions, but fewer colonies on solid mediums after reproduction in pH 6 and 7 conditions. These results demonstrate the antibacterial properties of the Mat@CS-Pro performance become worse as the pH value of the environment increases. Combined with the results in Figures 9B,C, the nanocapsules exhibit better antibacterial performance because the size of the nanocapsules is larger, and it is easier to release matrine in an acid environment.

## Conclusion

In this study, the Mat@CS-Pro nanocapsules with pH-responsive antibacterial performance and biofilm dispersibility were synthesized successfully. The Mat@CS nanocapsules were prepared initially *via* the microemulsion method, followed by immobilized D-proline on their surfaces. The coated D-proline cannot restrain the matrine releasing and Mat@CS-Pro nanocapsules maintained outstanding antibacterial properties of *E. coli, S. aureus*, and *P. aeruginosa*. After introducing D-proline on the Mat@CS surface, such nanocapsules exhibited biofilm inhibition properties, especially for gram-negative bacterial strains, with a reduction of up to 74% after incubation for 3 days. Besides, the Mat@CS-Pro nanocapsules had remarkable pH-responsive properties, swelling under acid conditions and shrinking under alkaline conditions. Due to the structural changes, the releasing behaviors of matrine were stronger with the environmental pH transforming from alkaline to acidic and exhibited outstanding antibacterial properties under an acidic environment. These nanocapsules provide a novel idea of designing pH-responsive antibacterial synergy with biofilm dispersion performance antifoulants.

## Data availability statement

The original contributions presented in the study are included in the article/supplementary materials, further inquiries can be directed to the corresponding authors.

## Author contributions

XH: investigation, methodology, and writing—original draft. WY: investigation and methodology. JY, YB, HQ, and YL: investigation. PJ: conceptualization and writing—review and editing. DZ: supervision, conceptualization, methodology, and writing—review and editing. All authors contributed to the discussion of the study.

## Funding

The study was supported by the China Postdoctoral Science Foundation (2021M700381), the Postdoctor Research Foundation of Shunde Graduate School of University of Science and Technology Beijing (2021BH003), and the Program of Shanghai Academic/Technology Research Leader (No. 20XD1431400).

## Conflict of interest

Author DZ was employed by Shanghai Aerospace Equipment Manufacturer. The remaining authors declare that the research was conducted in the absence of any commercial or financial relationships that could be construed as a potential conflict of interest.

## Publisher's note

All claims expressed in this article are solely those of the authors and do not necessarily represent those of their affiliated organizations, or those of the publisher, the editors and the reviewers. Any product that may be evaluated in this article, or claim that may be made by its manufacturer, is not guaranteed or endorsed by the publisher.
